# Hunterian Lectures on Tension, Inflammation of Bone, and on Cranial and Intra-Cranial Injuries

**Published:** 1889-03

**Authors:** 


*Hunterian Lectures on Tension, Inflammation of Bone, and on Cranial and Intra-cranial Injuries.* By T. BRYANT, F.R.C.S. Pp. 146. London: J. & A. CHURCHILL. 1888.

If there is not much of novelty in this small work, it is all truth, and important truth that bears repetition and enforcement. Sound clinical precepts, supported by honest and enlightened practice, are by no means the least worthy means of perpetuating the memory of the great man in whose honour these lectures were delivered.

